# Penta­carbonyl-1κ^2^ *C*,2κ^3^ *C*-[(diphenyl­phosphor­yl)diphenyl­phosphane-1κ*P*]-μ-ethane-1,2-dithiol­ato-1:2κ^4^ *S*,*S*′:*S*,*S*′-diiron(I)(*Fe—Fe*)

**DOI:** 10.1107/S1600536811042139

**Published:** 2011-10-22

**Authors:** Xu-Feng Liu, Xiao-Yong Yu

**Affiliations:** a Department of Chemical Engineering, Ningbo University of Technology, Ningbo 315016, People’s Republic of China

## Abstract

The dinuclear title compound, [Fe_2_(C_2_H_4_S_2_)(C_24_H_20_OP_2_)(CO)_5_] or (μ-SCH_2_CH_2_S-μ)Fe_2_(CO)_5_[Ph_2_PP(O)Ph_2_], con­tains a butterfly-shaped Fe_2_S_2_ core in which the Fe⋯Fe separation is 2.5275 (6) Å. One of the Fe atoms is also coordinated to three carbonyl ligands and the other to two carbonyl ligands and one phosphane ligand [Ph_2_PP(O)Ph_2_]. Both Fe-atom geometries could be described as grossly distorted octa­hedral and the Ph_2_PP(O)Ph_2_ ligand lies *trans* to the Fe⋯Fe link.

## Related literature

For more details about diiron dithiol­ate complexes, see: Song *et al.* (2005 ▶); Wang *et al.* (2009 ▶); Yin *et al.* (2011 ▶). 
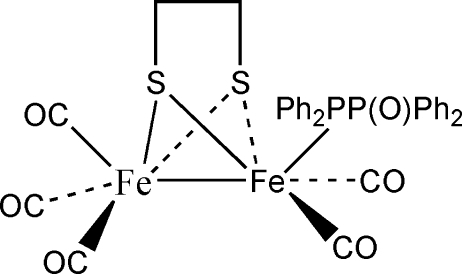


## Experimental

### 

#### Crystal data

[Fe_2_(C_2_H_4_S_2_)(C_24_H_20_OP_2_)(CO)_5_]
*M* *_r_* = 730.26
Monoclinic, 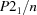

*a* = 13.865 (4) Å
*b* = 15.398 (4) Å
*c* = 14.459 (5) Å
β = 98.357 (4)°
*V* = 3054.1 (16) Å^3^
*Z* = 4
Mo *K*α radiation
μ = 1.24 mm^−1^
*T* = 113 K
0.20 × 0.18 × 0.10 mm

#### Data collection

Rigaku Saturn724 CCD diffractometer
Absorption correction: multi-scan (*CrystalClear*; Rigaku/MSC, 2005 ▶) *T*_min_ = 0.790, *T*_max_ = 0.886
31351 measured reflections
7281 independent reflections
6102 reflections with *I* > 2σ(*I*)
*R*_int_ = 0.039

#### Refinement

*R*[*F* ^2^ > 2σ(*F* ^2^)] = 0.026
*wR*(*F* ^2^) = 0.059
*S* = 1.06
7281 reflections
388 parameters
H-atom parameters constrained
Δρ_max_ = 0.36 e Å^−3^
Δρ_min_ = −0.36 e Å^−3^

### 

Data collection: *CrystalClear* (Rigaku/MSC, 2005 ▶); cell refinement: *CrystalClear*; data reduction: *CrystalClear*; program(s) used to solve structure: *SHELXS97* (Sheldrick, 2008 ▶); program(s) used to refine structure: *SHELXL97* (Sheldrick, 2008 ▶); molecular graphics: *SHELXTL* (Sheldrick, 2008 ▶); software used to prepare material for publication: *CrystalStructure* (Rigaku/MSC, 2005 ▶).

## Supplementary Material

Crystal structure: contains datablock(s) global, I. DOI: 10.1107/S1600536811042139/hb6442sup1.cif

Structure factors: contains datablock(s) I. DOI: 10.1107/S1600536811042139/hb6442Isup2.hkl

Additional supplementary materials:  crystallographic information; 3D view; checkCIF report

## Figures and Tables

**Table 1:** Selected bond lengths (Å)

Fe1—C2	1.7855 (18)
Fe1—C1	1.7981 (17)
Fe1—C3	1.8006 (18)
Fe1—S1	2.2484 (6)
Fe1—S2	2.2495 (8)
Fe2—C4	1.7733 (17)
Fe2—C5	1.7742 (17)
Fe2—P1	2.2426 (7)
Fe2—S1	2.2495 (7)
Fe2—S2	2.2530 (7)

**Fig. 1.**
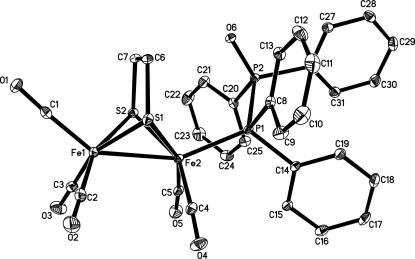
The molecular structure of (I). Displacement ellipsoids are drawn at the 30% probability level and H atoms are shown as small spheres of arbitrary radii.

**Fig. 2.**
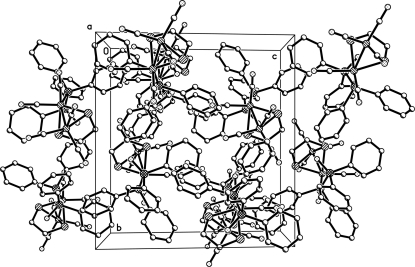
The crystal packing for (I).

## supplementary crystallographic information

### Comment

Diiron dithiolate complexes have received much attention in recent years due to their structures close to the active site of [FeFe]-hydrogenases (Song *et al.* (2005), Wang *et al.* (2009), Yin *et al.* (2011)). In continuation of our work in this area, the title complex, (I), was synthesized and its strucuture was determined by X-ray crstallography.

As shown in Fig. 1, the title complex contains five carbonyls and one Ph_2_PP(O)Ph_2_ ligands. The diiron ethanedithiolate cluster consists of two fused five-membered rings. The Ph_2_PP(O)Ph_2_ ligands occupies an axial position of the square-pyramidal geometry of the Fe atom.

### Experimental

The title complex was prepared from (µ-SCH_2_CH_2_S-µ)Fe_2_(CO)_6_ and 1,2-bis(diphenylphosphino)cyclopentane in the presence of Me_3_NO. Colourless prisms were grown from slow evaporation of dichloromethane and hexane solution at room temperature.

### Refinement

All the H atoms were positioned geometrically (C—H = 0.93–0.97 Å) and refined as riding with *U*_iso_(H) = 1.2*U*_eq_(C) or 1.5*U*_eq_(methyl C).

### Crystal data

**Table:**

[Fe_2_(C_2_H_4_S_2_)(C_24_H_20_OP_2_)(CO)_5_]	*F*(000) = 1488
*M**_r_* = 730.26	*D*_x_ = 1.588 Mg m^−^^3^
Monoclinic, *P*2_1_/*n*	Mo *K*α radiation, λ = 0.71073 Å
Hall symbol: -P 2yn	Cell parameters from 11006 reflections
*a* = 13.865 (4) Å	θ = 1.4–27.9°
*b* = 15.398 (4) Å	µ = 1.24 mm^−^^1^
*c* = 14.459 (5) Å	*T* = 113 K
β = 98.357 (4)°	Prism, colorless
*V* = 3054.1 (16) Å^3^	0.20 × 0.18 × 0.10 mm
*Z* = 4	

### Data collection

**Table:**

Rigaku Saturn724 CCD diffractometer	7281 independent reflections
Radiation source: rotating anode	6102 reflections with *I* > 2σ(*I*)
multilayer	*R*_int_ = 0.039
Detector resolution: 14.22 pixels mm^-1^	θ_max_ = 27.9°, θ_min_ = 1.9°
ω and φ scans	*h* = −18→18
Absorption correction: multi-scan (*CrystalClear*; Rigaku/MSC, 2005)	*k* = −17→20
*T*_min_ = 0.790, *T*_max_ = 0.886	*l* = −19→19
31351 measured reflections	

### Refinement

**Table:**

Refinement on *F*^2^	Primary atom site location: structure-invariant direct methods
Least-squares matrix: full	Secondary atom site location: difference Fourier map
*R*[*F*^2^ > 2σ(*F*^2^)] = 0.026	Hydrogen site location: inferred from neighbouring sites
*wR*(*F*^2^) = 0.059	H-atom parameters constrained
*S* = 1.06	*w* = 1/[σ^2^(*F*_o_^2^) + (0.024*P*)^2^] where *P* = (*F*_o_^2^ + 2*F*_c_^2^)/3
7281 reflections	(Δ/σ)_max_ = 0.002
388 parameters	Δρ_max_ = 0.36 e Å^−^^3^
0 restraints	Δρ_min_ = −0.36 e Å^−^^3^

### Special details

**Table:**

Geometry. All e.s.d.'s (except the e.s.d. in the dihedral angle between two l.s. planes) are estimated using the full covariance matrix. The cell e.s.d.'s are taken into account individually in the estimation of e.s.d.'s in distances, angles and torsion angles; correlations between e.s.d.'s in cell parameters are only used when they are defined by crystal symmetry. An approximate (isotropic) treatment of cell e.s.d.'s is used for estimating e.s.d.'s involving l.s. planes.
Refinement. Refinement of *F*^2^ against ALL reflections. The weighted *R*-factor *wR* and goodness of fit *S* are based on *F*^2^, conventional *R*-factors *R* are based on *F*, with *F* set to zero for negative *F*^2^. The threshold expression of *F*^2^ > σ(*F*^2^) is used only for calculating *R*-factors(gt) *etc*. and is not relevant to the choice of reflections for refinement. *R*-factors based on *F*^2^ are statistically about twice as large as those based on *F*, and *R*- factors based on ALL data will be even larger.

### Fractional atomic coordinates and isotropic or equivalent isotropic displacement parameters (Å^2^)

**Table:**

	*x*	*y*	*z*	*U*_iso_*/*U*_eq_	
Fe1	1.261528 (16)	0.058771 (14)	0.331352 (16)	0.01557 (6)	
Fe2	1.133418 (15)	0.174298 (14)	0.288051 (14)	0.01275 (6)	
P1	0.99707 (3)	0.24364 (3)	0.31226 (3)	0.01291 (9)	
P2	0.86096 (3)	0.16331 (3)	0.27704 (3)	0.01404 (9)	
S1	1.18853 (3)	0.13327 (3)	0.43559 (3)	0.01697 (9)	
S2	1.10474 (3)	0.03046 (2)	0.27533 (3)	0.01596 (9)	
O1	1.30120 (9)	−0.10606 (7)	0.43282 (8)	0.0300 (3)	
O2	1.43873 (9)	0.15892 (8)	0.39375 (10)	0.0411 (4)	
O3	1.32473 (9)	0.01325 (8)	0.15188 (9)	0.0366 (3)	
O4	1.26144 (9)	0.32544 (8)	0.29575 (9)	0.0321 (3)	
O5	1.12828 (9)	0.17617 (8)	0.08534 (8)	0.0298 (3)	
O6	0.86136 (7)	0.09157 (6)	0.34670 (7)	0.0177 (2)	
C1	1.28842 (11)	−0.04173 (11)	0.39306 (11)	0.0209 (4)	
C2	1.36981 (12)	0.11973 (11)	0.36834 (13)	0.0250 (4)	
C3	1.29927 (12)	0.03076 (10)	0.22113 (12)	0.0232 (4)	
C4	1.20985 (12)	0.26713 (10)	0.29551 (11)	0.0193 (3)	
C5	1.12641 (11)	0.17650 (10)	0.16457 (11)	0.0185 (3)	
C6	1.09760 (11)	0.05588 (10)	0.46439 (11)	0.0209 (4)	
H6A	1.1266	0.0200	0.5182	0.025*	
H6B	1.0418	0.0881	0.4833	0.025*	
C7	1.06081 (12)	−0.00340 (10)	0.38252 (11)	0.0195 (3)	
H7A	0.9886	−0.0031	0.3724	0.023*	
H7B	1.0827	−0.0636	0.3979	0.023*	
C8	0.99500 (11)	0.27904 (10)	0.43317 (10)	0.0167 (3)	
C9	1.05975 (13)	0.34539 (11)	0.46564 (12)	0.0259 (4)	
H9	1.1029	0.3676	0.4260	0.031*	
C10	1.06217 (14)	0.37937 (12)	0.55452 (12)	0.0328 (4)	
H10	1.1068	0.4244	0.5756	0.039*	
C11	0.99951 (13)	0.34765 (12)	0.61251 (12)	0.0310 (4)	
H11	1.0001	0.3716	0.6731	0.037*	
C12	0.93619 (13)	0.28118 (12)	0.58215 (12)	0.0303 (4)	
H12	0.8940	0.2589	0.6226	0.036*	
C13	0.93332 (11)	0.24635 (11)	0.49297 (11)	0.0209 (4)	
H13	0.8895	0.2004	0.4729	0.025*	
C14	0.96328 (11)	0.34554 (9)	0.24976 (11)	0.0151 (3)	
C15	1.00002 (11)	0.36534 (10)	0.16773 (11)	0.0186 (3)	
H15	1.0464	0.3279	0.1462	0.022*	
C16	0.96935 (12)	0.43952 (10)	0.11709 (12)	0.0247 (4)	
H16	0.9938	0.4517	0.0604	0.030*	
C17	0.90373 (12)	0.49556 (11)	0.14854 (12)	0.0258 (4)	
H17	0.8830	0.5462	0.1137	0.031*	
C18	0.86815 (12)	0.47762 (11)	0.23126 (12)	0.0254 (4)	
H18	0.8233	0.5163	0.2535	0.031*	
C19	0.89793 (11)	0.40335 (10)	0.28154 (11)	0.0210 (4)	
H19	0.8735	0.3916	0.3384	0.025*	
C20	0.86646 (11)	0.12448 (10)	0.16014 (10)	0.0167 (3)	
C21	0.85652 (12)	0.03529 (10)	0.14617 (11)	0.0235 (4)	
H21	0.8466	−0.0013	0.1968	0.028*	
C22	0.86095 (14)	−0.00041 (12)	0.05905 (12)	0.0327 (4)	
H22	0.8525	−0.0612	0.0498	0.039*	
C23	0.87764 (13)	0.05197 (12)	−0.01452 (12)	0.0308 (4)	
H23	0.8817	0.0272	−0.0740	0.037*	
C24	0.88851 (12)	0.14079 (12)	−0.00128 (12)	0.0257 (4)	
H24	0.9003	0.1769	−0.0517	0.031*	
C25	0.88221 (11)	0.17707 (11)	0.08506 (11)	0.0214 (4)	
H25	0.8886	0.2381	0.0934	0.026*	
C26	0.75306 (11)	0.22975 (10)	0.27313 (10)	0.0156 (3)	
C27	0.70310 (11)	0.22562 (10)	0.35030 (11)	0.0185 (3)	
H27	0.7270	0.1897	0.4020	0.022*	
C28	0.61916 (11)	0.27358 (10)	0.35174 (12)	0.0224 (4)	
H28	0.5855	0.2704	0.4043	0.027*	
C29	0.58382 (12)	0.32645 (10)	0.27659 (12)	0.0252 (4)	
H29	0.5263	0.3597	0.2777	0.030*	
C30	0.63304 (12)	0.33036 (11)	0.20009 (12)	0.0265 (4)	
H30	0.6088	0.3662	0.1485	0.032*	
C31	0.71692 (11)	0.28270 (10)	0.19801 (11)	0.0216 (4)	
H31	0.7502	0.2860	0.1451	0.026*	

### Atomic displacement parameters (Å^2^)

**Table:**

	*U*^11^	*U*^22^	*U*^33^	*U*^12^	*U*^13^	*U*^23^
Fe1	0.01547 (12)	0.01379 (12)	0.01783 (12)	0.00070 (9)	0.00372 (9)	0.00033 (9)
Fe2	0.01568 (12)	0.01181 (12)	0.01079 (11)	−0.00046 (8)	0.00202 (8)	0.00040 (8)
P1	0.0163 (2)	0.0118 (2)	0.01058 (19)	−0.00003 (15)	0.00202 (15)	0.00057 (15)
P2	0.0160 (2)	0.0135 (2)	0.01251 (19)	−0.00015 (15)	0.00168 (15)	0.00074 (15)
S1	0.0216 (2)	0.0164 (2)	0.01245 (19)	0.00252 (16)	0.00080 (15)	0.00018 (15)
S2	0.0180 (2)	0.0133 (2)	0.0166 (2)	−0.00165 (15)	0.00262 (15)	−0.00067 (15)
O1	0.0367 (7)	0.0210 (7)	0.0332 (7)	0.0076 (5)	0.0084 (6)	0.0089 (6)
O2	0.0249 (7)	0.0301 (8)	0.0672 (11)	−0.0091 (6)	0.0033 (7)	−0.0059 (7)
O3	0.0429 (8)	0.0386 (8)	0.0332 (8)	−0.0044 (6)	0.0223 (7)	−0.0082 (6)
O4	0.0317 (7)	0.0245 (7)	0.0378 (8)	−0.0127 (6)	−0.0031 (6)	0.0043 (6)
O5	0.0450 (8)	0.0307 (7)	0.0149 (6)	0.0051 (6)	0.0088 (6)	0.0003 (5)
O6	0.0208 (6)	0.0153 (6)	0.0174 (6)	−0.0001 (4)	0.0044 (5)	0.0040 (5)
C1	0.0178 (9)	0.0233 (10)	0.0224 (9)	0.0015 (7)	0.0059 (7)	−0.0028 (7)
C2	0.0224 (10)	0.0193 (9)	0.0341 (10)	0.0032 (7)	0.0069 (8)	0.0000 (8)
C3	0.0235 (9)	0.0171 (9)	0.0303 (10)	−0.0024 (7)	0.0083 (8)	0.0009 (7)
C4	0.0229 (9)	0.0187 (9)	0.0152 (8)	0.0021 (7)	−0.0007 (6)	0.0022 (7)
C5	0.0231 (9)	0.0126 (8)	0.0201 (9)	0.0006 (6)	0.0038 (7)	0.0004 (7)
C6	0.0242 (9)	0.0216 (9)	0.0190 (9)	0.0043 (7)	0.0100 (7)	0.0081 (7)
C7	0.0198 (9)	0.0160 (9)	0.0241 (9)	−0.0004 (6)	0.0083 (7)	0.0062 (7)
C8	0.0216 (8)	0.0162 (8)	0.0117 (8)	0.0048 (6)	−0.0002 (6)	−0.0015 (6)
C9	0.0349 (10)	0.0230 (9)	0.0198 (9)	−0.0062 (7)	0.0040 (7)	−0.0029 (7)
C10	0.0465 (12)	0.0260 (10)	0.0241 (10)	−0.0026 (8)	−0.0007 (8)	−0.0094 (8)
C11	0.0419 (12)	0.0354 (11)	0.0147 (9)	0.0088 (9)	0.0010 (8)	−0.0093 (8)
C12	0.0296 (10)	0.0450 (12)	0.0175 (9)	0.0072 (8)	0.0078 (7)	−0.0010 (8)
C13	0.0216 (9)	0.0253 (10)	0.0158 (8)	0.0043 (7)	0.0023 (7)	−0.0009 (7)
C14	0.0170 (8)	0.0127 (8)	0.0148 (8)	−0.0018 (6)	−0.0008 (6)	0.0007 (6)
C15	0.0203 (9)	0.0150 (8)	0.0205 (8)	−0.0011 (6)	0.0032 (7)	0.0012 (7)
C16	0.0294 (10)	0.0226 (9)	0.0215 (9)	−0.0025 (7)	0.0014 (7)	0.0079 (7)
C17	0.0284 (10)	0.0150 (9)	0.0312 (10)	0.0007 (7)	−0.0050 (8)	0.0072 (7)
C18	0.0252 (10)	0.0165 (9)	0.0336 (10)	0.0052 (7)	0.0007 (8)	−0.0004 (7)
C19	0.0239 (9)	0.0174 (9)	0.0219 (9)	0.0002 (7)	0.0040 (7)	0.0006 (7)
C20	0.0152 (8)	0.0194 (9)	0.0150 (8)	0.0009 (6)	0.0005 (6)	−0.0022 (6)
C21	0.0318 (10)	0.0195 (9)	0.0186 (9)	0.0011 (7)	0.0021 (7)	−0.0014 (7)
C22	0.0524 (13)	0.0205 (10)	0.0243 (10)	0.0032 (8)	0.0018 (9)	−0.0067 (8)
C23	0.0390 (11)	0.0365 (11)	0.0164 (9)	0.0011 (8)	0.0022 (8)	−0.0093 (8)
C24	0.0270 (10)	0.0329 (11)	0.0170 (9)	−0.0052 (8)	0.0028 (7)	−0.0006 (7)
C25	0.0240 (9)	0.0211 (9)	0.0186 (8)	−0.0040 (7)	0.0012 (7)	−0.0015 (7)
C26	0.0156 (8)	0.0138 (8)	0.0171 (8)	−0.0016 (6)	0.0011 (6)	−0.0003 (6)
C27	0.0192 (8)	0.0180 (9)	0.0179 (8)	−0.0032 (6)	0.0018 (6)	0.0001 (7)
C28	0.0196 (9)	0.0245 (9)	0.0239 (9)	−0.0035 (7)	0.0057 (7)	−0.0054 (7)
C29	0.0183 (9)	0.0213 (9)	0.0348 (10)	0.0037 (7)	0.0002 (7)	−0.0053 (8)
C30	0.0247 (9)	0.0248 (10)	0.0282 (10)	0.0065 (7)	−0.0019 (8)	0.0062 (8)
C31	0.0220 (9)	0.0231 (9)	0.0197 (9)	0.0006 (7)	0.0031 (7)	0.0022 (7)

### Geometric parameters (Å, °)

**Table:**

Fe1—C2	1.7855 (18)	C12—C13	1.392 (2)
Fe1—C1	1.7981 (17)	C12—H12	0.9500
Fe1—C3	1.8006 (18)	C13—H13	0.9500
Fe1—S1	2.2484 (6)	C14—C15	1.391 (2)
Fe1—S2	2.2495 (8)	C14—C19	1.395 (2)
Fe1—Fe2	2.5275 (6)	C15—C16	1.390 (2)
Fe2—C4	1.7733 (17)	C15—H15	0.9500
Fe2—C5	1.7742 (17)	C16—C17	1.378 (2)
Fe2—P1	2.2426 (7)	C16—H16	0.9500
Fe2—S1	2.2495 (7)	C17—C18	1.386 (2)
Fe2—S2	2.2530 (7)	C17—H17	0.9500
P1—C8	1.8353 (16)	C18—C19	1.386 (2)
P1—C14	1.8371 (16)	C18—H18	0.9500
P1—P2	2.2519 (8)	C19—H19	0.9500
P2—O6	1.4943 (11)	C20—C21	1.392 (2)
P2—C20	1.8049 (16)	C20—C25	1.397 (2)
P2—C26	1.8066 (16)	C21—C22	1.384 (2)
S1—C6	1.8264 (16)	C21—H21	0.9500
S2—C7	1.8219 (16)	C22—C23	1.381 (2)
O1—C1	1.1461 (19)	C22—H22	0.9500
O2—C2	1.145 (2)	C23—C24	1.386 (2)
O3—C3	1.1414 (19)	C23—H23	0.9500
O4—C4	1.1476 (18)	C24—C25	1.382 (2)
O5—C5	1.1496 (19)	C24—H24	0.9500
C6—C7	1.523 (2)	C25—H25	0.9500
C6—H6A	0.9900	C26—C31	1.392 (2)
C6—H6B	0.9900	C26—C27	1.398 (2)
C7—H7A	0.9900	C27—C28	1.381 (2)
C7—H7B	0.9900	C27—H27	0.9500
C8—C9	1.396 (2)	C28—C29	1.389 (2)
C8—C13	1.396 (2)	C28—H28	0.9500
C9—C10	1.383 (2)	C29—C30	1.383 (2)
C9—H9	0.9500	C29—H29	0.9500
C10—C11	1.381 (2)	C30—C31	1.379 (2)
C10—H10	0.9500	C30—H30	0.9500
C11—C12	1.378 (3)	C31—H31	0.9500
C11—H11	0.9500		
			
C2—Fe1—C1	101.48 (8)	C10—C9—H9	119.4
C2—Fe1—C3	92.93 (8)	C8—C9—H9	119.4
C1—Fe1—C3	99.62 (7)	C11—C10—C9	119.85 (17)
C2—Fe1—S1	88.39 (6)	C11—C10—H10	120.1
C1—Fe1—S1	100.81 (5)	C9—C10—H10	120.1
C3—Fe1—S1	158.84 (6)	C12—C11—C10	119.80 (16)
C2—Fe1—S2	159.44 (5)	C12—C11—H11	120.1
C1—Fe1—S2	97.57 (5)	C10—C11—H11	120.1
C3—Fe1—S2	91.43 (6)	C11—C12—C13	120.83 (16)
S1—Fe1—S2	80.48 (2)	C11—C12—H12	119.6
C2—Fe1—Fe2	103.55 (6)	C13—C12—H12	119.6
C1—Fe1—Fe2	144.71 (5)	C12—C13—C8	119.83 (16)
C3—Fe1—Fe2	103.51 (6)	C12—C13—H13	120.1
S1—Fe1—Fe2	55.831 (19)	C8—C13—H13	120.1
S2—Fe1—Fe2	55.91 (2)	C15—C14—C19	118.50 (14)
C4—Fe2—C5	89.47 (7)	C15—C14—P1	120.51 (11)
C4—Fe2—P1	96.70 (6)	C19—C14—P1	120.96 (12)
C5—Fe2—P1	102.94 (5)	C16—C15—C14	120.49 (15)
C4—Fe2—S1	92.92 (5)	C16—C15—H15	119.8
C5—Fe2—S1	157.08 (5)	C14—C15—H15	119.8
P1—Fe2—S1	99.41 (2)	C17—C16—C15	120.46 (16)
C4—Fe2—S2	153.40 (5)	C17—C16—H16	119.8
C5—Fe2—S2	87.33 (5)	C15—C16—H16	119.8
P1—Fe2—S2	109.75 (2)	C16—C17—C18	119.65 (15)
S1—Fe2—S2	80.385 (18)	C16—C17—H17	120.2
C4—Fe2—Fe1	99.20 (6)	C18—C17—H17	120.2
C5—Fe2—Fe1	101.33 (5)	C19—C18—C17	120.06 (15)
P1—Fe2—Fe1	150.983 (16)	C19—C18—H18	120.0
S1—Fe2—Fe1	55.791 (13)	C17—C18—H18	120.0
S2—Fe2—Fe1	55.79 (2)	C18—C19—C14	120.81 (15)
C8—P1—C14	100.18 (7)	C18—C19—H19	119.6
C8—P1—Fe2	114.85 (5)	C14—C19—H19	119.6
C14—P1—Fe2	119.36 (5)	C21—C20—C25	118.86 (14)
C8—P1—P2	104.48 (5)	C21—C20—P2	116.48 (12)
C14—P1—P2	102.27 (5)	C25—C20—P2	124.64 (12)
Fe2—P1—P2	113.64 (3)	C22—C21—C20	120.49 (16)
O6—P2—C20	112.94 (7)	C22—C21—H21	119.8
O6—P2—C26	111.20 (6)	C20—C21—H21	119.8
C20—P2—C26	107.80 (7)	C23—C22—C21	120.24 (17)
O6—P2—P1	109.43 (5)	C23—C22—H22	119.9
C20—P2—P1	104.22 (5)	C21—C22—H22	119.9
C26—P2—P1	111.04 (6)	C22—C23—C24	119.80 (16)
C6—S1—Fe1	102.29 (6)	C22—C23—H23	120.1
C6—S1—Fe2	104.41 (6)	C24—C23—H23	120.1
Fe1—S1—Fe2	68.38 (2)	C25—C24—C23	120.25 (16)
C7—S2—Fe1	100.03 (6)	C25—C24—H24	119.9
C7—S2—Fe2	106.78 (5)	C23—C24—H24	119.9
Fe1—S2—Fe2	68.299 (13)	C24—C25—C20	120.35 (16)
O1—C1—Fe1	176.91 (14)	C24—C25—H25	119.8
O2—C2—Fe1	178.68 (16)	C20—C25—H25	119.8
O3—C3—Fe1	178.89 (16)	C31—C26—C27	119.05 (14)
O4—C4—Fe2	175.95 (15)	C31—C26—P2	123.97 (12)
O5—C5—Fe2	175.41 (15)	C27—C26—P2	116.97 (12)
C7—C6—S1	112.22 (10)	C28—C27—C26	120.28 (15)
C7—C6—H6A	109.2	C28—C27—H27	119.9
S1—C6—H6A	109.2	C26—C27—H27	119.9
C7—C6—H6B	109.2	C27—C28—C29	120.26 (15)
S1—C6—H6B	109.2	C27—C28—H28	119.9
H6A—C6—H6B	107.9	C29—C28—H28	119.9
C6—C7—S2	111.97 (10)	C30—C29—C28	119.49 (15)
C6—C7—H7A	109.2	C30—C29—H29	120.3
S2—C7—H7A	109.2	C28—C29—H29	120.3
C6—C7—H7B	109.2	C31—C30—C29	120.67 (16)
S2—C7—H7B	109.2	C31—C30—H30	119.7
H7A—C7—H7B	107.9	C29—C30—H30	119.7
C9—C8—C13	118.54 (14)	C30—C31—C26	120.25 (15)
C9—C8—P1	116.08 (12)	C30—C31—H31	119.9
C13—C8—P1	125.37 (12)	C26—C31—H31	119.9
C10—C9—C8	121.13 (16)		
			
C2—Fe1—Fe2—C4	8.72 (8)	C5—Fe2—S2—C7	−160.02 (8)
C1—Fe1—Fe2—C4	142.75 (11)	P1—Fe2—S2—C7	−57.23 (6)
C3—Fe1—Fe2—C4	−87.70 (7)	S1—Fe2—S2—C7	39.40 (6)
S1—Fe1—Fe2—C4	87.27 (5)	Fe1—Fe2—S2—C7	94.35 (6)
S2—Fe1—Fe2—C4	−170.14 (5)	C4—Fe2—S2—Fe1	22.18 (11)
C2—Fe1—Fe2—C5	100.03 (8)	C5—Fe2—S2—Fe1	105.64 (5)
C1—Fe1—Fe2—C5	−125.94 (11)	P1—Fe2—S2—Fe1	−151.57 (2)
C3—Fe1—Fe2—C5	3.60 (7)	S1—Fe2—S2—Fe1	−54.945 (14)
S1—Fe1—Fe2—C5	178.57 (5)	C4—Fe2—C5—O5	70.8 (18)
S2—Fe1—Fe2—C5	−78.83 (5)	P1—Fe2—C5—O5	167.5 (18)
C2—Fe1—Fe2—P1	−113.67 (7)	S1—Fe2—C5—O5	−25.5 (19)
C1—Fe1—Fe2—P1	20.35 (10)	S2—Fe2—C5—O5	−82.8 (18)
C3—Fe1—Fe2—P1	149.90 (6)	Fe1—Fe2—C5—O5	−28.5 (18)
S1—Fe1—Fe2—P1	−35.13 (3)	Fe1—S1—C6—C7	−31.06 (12)
S2—Fe1—Fe2—P1	67.46 (4)	Fe2—S1—C6—C7	39.46 (12)
C2—Fe1—Fe2—S1	−78.54 (6)	S1—C6—C7—S2	−8.36 (15)
C1—Fe1—Fe2—S1	55.48 (9)	Fe1—S2—C7—C6	43.88 (11)
C3—Fe1—Fe2—S1	−174.97 (6)	Fe2—S2—C7—C6	−26.31 (12)
S2—Fe1—Fe2—S1	102.59 (2)	C14—P1—C8—C9	−60.76 (14)
C2—Fe1—Fe2—S2	178.86 (6)	Fe2—P1—C8—C9	68.45 (13)
C1—Fe1—Fe2—S2	−47.11 (9)	P2—P1—C8—C9	−166.39 (12)
C3—Fe1—Fe2—S2	82.44 (5)	C14—P1—C8—C13	117.87 (14)
S1—Fe1—Fe2—S2	−102.59 (2)	Fe2—P1—C8—C13	−112.92 (13)
C4—Fe2—P1—C8	−73.60 (8)	P2—P1—C8—C13	12.24 (14)
C5—Fe2—P1—C8	−164.59 (8)	C13—C8—C9—C10	−1.0 (3)
S1—Fe2—P1—C8	20.50 (6)	P1—C8—C9—C10	177.70 (14)
S2—Fe2—P1—C8	103.59 (6)	C8—C9—C10—C11	−0.2 (3)
Fe1—Fe2—P1—C8	49.34 (7)	C9—C10—C11—C12	1.2 (3)
C4—Fe2—P1—C14	45.35 (8)	C10—C11—C12—C13	−1.1 (3)
C5—Fe2—P1—C14	−45.64 (8)	C11—C12—C13—C8	−0.2 (3)
S1—Fe2—P1—C14	139.45 (6)	C9—C8—C13—C12	1.2 (2)
S2—Fe2—P1—C14	−137.46 (6)	P1—C8—C13—C12	−177.41 (13)
Fe1—Fe2—P1—C14	168.29 (6)	C8—P1—C14—C15	147.58 (13)
C4—Fe2—P1—P2	166.17 (5)	Fe2—P1—C14—C15	21.35 (15)
C5—Fe2—P1—P2	75.18 (5)	P2—P1—C14—C15	−105.03 (13)
S1—Fe2—P1—P2	−99.72 (2)	C8—P1—C14—C19	−34.57 (14)
S2—Fe2—P1—P2	−16.64 (3)	Fe2—P1—C14—C19	−160.80 (11)
Fe1—Fe2—P1—P2	−70.88 (4)	P2—P1—C14—C19	72.82 (13)
C8—P1—P2—O6	−56.30 (7)	C19—C14—C15—C16	−2.3 (2)
C14—P1—P2—O6	−160.36 (6)	P1—C14—C15—C16	175.62 (12)
Fe2—P1—P2—O6	69.63 (5)	C14—C15—C16—C17	1.5 (2)
C8—P1—P2—C20	−177.36 (7)	C15—C16—C17—C18	0.0 (3)
C14—P1—P2—C20	78.58 (7)	C16—C17—C18—C19	−0.5 (3)
Fe2—P1—P2—C20	−51.43 (6)	C17—C18—C19—C14	−0.4 (3)
C8—P1—P2—C26	66.83 (7)	C15—C14—C19—C18	1.7 (2)
C14—P1—P2—C26	−37.23 (7)	P1—C14—C19—C18	−176.14 (13)
Fe2—P1—P2—C26	−167.24 (5)	O6—P2—C20—C21	8.73 (15)
C2—Fe1—S1—C6	−151.58 (8)	C26—P2—C20—C21	−114.53 (13)
C1—Fe1—S1—C6	−50.20 (8)	P1—P2—C20—C21	127.42 (12)
C3—Fe1—S1—C6	114.47 (16)	O6—P2—C20—C25	−169.59 (13)
S2—Fe1—S1—C6	45.77 (6)	C26—P2—C20—C25	67.16 (15)
Fe2—Fe1—S1—C6	100.81 (6)	P1—P2—C20—C25	−50.90 (14)
C2—Fe1—S1—Fe2	107.60 (6)	C25—C20—C21—C22	−0.8 (2)
C1—Fe1—S1—Fe2	−151.01 (5)	P2—C20—C21—C22	−179.23 (14)
C3—Fe1—S1—Fe2	13.65 (15)	C20—C21—C22—C23	1.6 (3)
S2—Fe1—S1—Fe2	−55.042 (18)	C21—C22—C23—C24	−1.0 (3)
C4—Fe2—S1—C6	163.12 (7)	C22—C23—C24—C25	−0.3 (3)
C5—Fe2—S1—C6	−101.33 (14)	C23—C24—C25—C20	1.1 (3)
P1—Fe2—S1—C6	65.83 (6)	C21—C20—C25—C24	−0.5 (2)
S2—Fe2—S1—C6	−42.79 (5)	P2—C20—C25—C24	177.80 (13)
Fe1—Fe2—S1—C6	−97.73 (6)	O6—P2—C26—C31	−159.14 (13)
C4—Fe2—S1—Fe1	−99.14 (6)	C20—P2—C26—C31	−34.83 (16)
C5—Fe2—S1—Fe1	−3.59 (13)	P1—P2—C26—C31	78.75 (14)
P1—Fe2—S1—Fe1	163.565 (15)	O6—P2—C26—C27	19.76 (14)
S2—Fe2—S1—Fe1	54.94 (2)	C20—P2—C26—C27	144.07 (12)
C2—Fe1—S2—C7	−107.34 (17)	P1—P2—C26—C27	−102.35 (12)
C1—Fe1—S2—C7	50.53 (7)	C31—C26—C27—C28	−0.1 (2)
C3—Fe1—S2—C7	150.43 (7)	P2—C26—C27—C28	−179.03 (12)
S1—Fe1—S2—C7	−49.23 (5)	C26—C27—C28—C29	−0.1 (2)
Fe2—Fe1—S2—C7	−104.19 (5)	C27—C28—C29—C30	0.3 (2)
C2—Fe1—S2—Fe2	−3.15 (16)	C28—C29—C30—C31	−0.3 (3)
C1—Fe1—S2—Fe2	154.72 (5)	C29—C30—C31—C26	0.1 (3)
C3—Fe1—S2—Fe2	−105.38 (5)	C27—C26—C31—C30	0.1 (2)
S1—Fe1—S2—Fe2	54.960 (17)	P2—C26—C31—C30	178.96 (13)
C4—Fe2—S2—C7	116.53 (12)		
